# Pharmacological Sphincterotomy for Chronic Anal Fissures by Botulinum Toxin A

**DOI:** 10.4103/0974-2077.44160

**Published:** 2008

**Authors:** Uwe Wollina

**Affiliations:** *Department of Dermatology and Allergology, Hospital Dresden-Friedrichstadt, Academic Teaching Hospital of the University of Dresden, Friedrichstrasse 41, Dresden, Germany.*

**Keywords:** Anal fissure, botulinum toxin, lateral sphincterotomy, therapeutic algorithm

## Abstract

Chronic anal fissure is a common proctologic disease. Botulinum toxin (BTX) can be used for temporary chemical denervation to treat this painful disorder. Its application is by intramuscular injections into either the external or internal anal sphincter muscle. The mode of action, application techniques, and possible complications or adverse effects of BTX therapy are discussed in this report. The healing rate is dependent on the BTX dosage. The short-term healing rate (≤ 6 months) is 60–90%, whereas about 50% of the patients show a complete response in long-term follow-up studies (> 1 year). Adverse effects are generally mild, but relapses occur more often than with surgery. Conservative therapy is currently considered as a first-line treatment. With increasing evidence for its efficacy, BTX can now be considered among the first-line nonsurgical treatements. Although, surgical management by lateral sphincterotomy is the most effective treatment, it shows a higher incidence of incontinence and greater general morbidity rate than BTX. BTX is a useful alternative to surgery and in many cases, surgery can be avoided with the use of BTX.

## ANAL FISSURES

**Figure 1:**
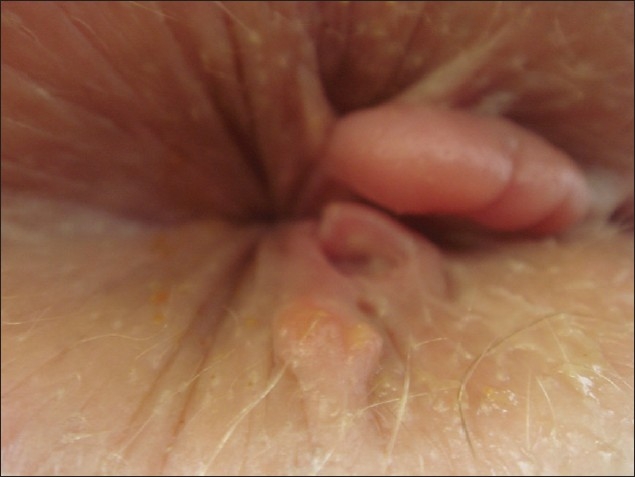
Chronic anal fissure and marisca

An anal fissure is a defect in the epithelium of the anal canal from the ano-cutaneous border to the linea dentata. Chronic fissures are characterised by a sentinel tag, hypertophic anal papillae, anal spasm, and/or fibrosis of the inner sphincter muscle [Figure 1]. Chronic fissures are commonly seen at 6 o’clock with the patient in a recumbent position; fissures at any other position need further investigation as to the underlying cause. Possible causes are Crohn’s disease, anal intercourse, sexually transmitted disease, or anal carcinoma [Table 1].[1,2]

**Table 1:** Diseases associated with chronic anal fissures

Chronic inflammatory gut diseases (Crohn’s disease, colitis ulcerosa)
Chronic infections (tuberculosis and other mycobacterial infections, HIV, syphilis)
Solid tumours (anal carcinoma and other carcinomas)
Haematologic diseases (leukaemia, lymphoma, plasmocytoma)
Chemo- or radiotherapy
Trauma

Relaxation of the sphincter internus can be inhibited by nonadrenergic, noncholinergic enteric neurons, parasympathetic muscarinic receptors, sympathetic beta receptors, or by inhibition of intracellular calcium uptake.[3]

Acute anal fissures are extremely painful during defecation; the pain is cramp-like and may persist for hours. With chronic anal fissures, the pain may be less intense during defecation, but increases after that. Perianal eczema is often associated with chronic anal fissures. Hyperhidrosis of the anal fold aggravates these symptoms.[4]

The aetiopathogenesis of the chronic anal fissure is not well understood. There is increased intraanal pressure at rest that might contribute to an ischemic state of the anal sphincter muscles. Indeed, the anodermal blood flow of the posterior midline has been shown to be reduced.[1] On the other hand, there is an increased local innervation.[5] One’s established a devil’s circle of spasm and pain develops.

## BOTULINUM TOXIN A

Botulinum toxin A (BTX A) is produced by *Clostridium botulinum* but can be synthesised as a single chain polypeptide of ~150 kDa that acts as a zinc-dependent endopeptidase. BTX A cleaves SNAP-25, a part of the SNARE complex that is responsible for acetylcholine transport from the nerve to the muscle end plate.

BTX action on hyperactive smooth muscles such as the anal sphincter is mediated by its action on the autonomic nervous system (as in striated muscles). The treatment goal for BTX is the interruption of the internal sphincter spasm and thereby, the ischemic state. Indeed, sphincter manometry after BTX injection has demonstrated a lowering of resting intranal pressure.[6]

## SELECTION OF PATIENTS

The diagnosis of a chronic anal fissure has to be established by proper examination and underlying diseases need appropriate therapy. None of the available BTX products on the market has been approved for the indication of anal fissures. Therefore, in its current status, this is an off-label use for BTX A. Hence, the patient should be properly counselled with an extensive verbal and written explanation, and his/her verbal and written consent obtained.

General contraindications for the use of BTX have to be considered before its use [Table 2].[7]

**Table 2:** Contraindications for botulinum toxin

**Absolute contraindications**
Gravidity and lactation
Haemophilia
Any drug treatment that increases the risk of bleeding
Myasthenia gravis
Lambert-Eaton syndrome
Amyotrophic lateral sclerosis
Known allergies against any of the ingredients of commercially
available BTX formulations (such as albumin), allergies against BTX
itself have yet not been reported.
Simultaneous or concommittent use of drugs such as aminoglycoside
antibiotics (streptomycin, kanamycin, gentamycin, neomycin,
spectinomycin etc.) since they might interfere with the metabolism of
BTX
**Relative contraindications**
Fluoride infections
Severe smokers (healing impaired, interference with clotting)
Noncompliance

BTX – Botulinum toxin

## PROCEDURE OF BTX ADMINISTRATION

BTX is available in a freeze-dried form. Published studies have been performed with either Botox® (Allergan) or Dysport® (Ipsen-Speywood). One Botox®-unit translates into two or three Dysport®-units. The vials have to be resolved with sterile physiological sodium chloride solution (according to the manufacturer’s instructions with 2–2.5 mL). Higher concentrations might be necessary in individual cases.

For injections, easy-to-use syringes such as the insulin syringes or those used for hyposensitization are preferred. They have a maximum capacity of 1 mL and markings that allow safe injections of volumes as low as 0.1 mL, which is the usual volume to inject per site. A fine and sharp needle of 27–32 gauge should be employed to minimize pain and bleeding.[2]

Injection should be done with the patient lying on one side or in a recumbent position. An intramuscular injection is performed after local disinfection. Injections into both the internal as well as the external sphincter muscles have been described in literature. For the former technique, the doctor has to put 2–3 fingers *in ano* for the exact localization of the needle. This technique causes more pain for the patient and also carries the risk of needle stick injury for the doctor as well. Therefore, sedation of the patient is often necessary. Injection into the external sphincter avoids these risks and sedation is not necessary. However, as the internal sphincter is also involved in the pathophysiology of anal fissures, injection into only the external sphincter may not be fully effective in releiving the spasm. However, BTX shows a three-dimensional diffusion of about 2 cm, which is considered adequate to reach the internal sphincter as well.[8]

The injections are usually placed along the fissure on both sides at a distance of 1–2 cm as fissure-associated ischaemia will prevent diffusion. In a recent study, Maria *et al*. demonstrated that a perifissural injection in the posterior-median localization is less effective than an injection along the anterior midline. They observed an increased relaxation effect by the latter technique.[9]

Different dosage schedules have been used. Most investors use higher dosages as the response rate is higher and the relapse rate is lower. Up to 100 U BTXA (Botox®) have been used without any severe adverse effects; the usual initial dosage is 20–40 U Botox® or 50–100 U Dysport®.[8]

## OUTCOME AND SAFETY: PUBLISHED STUDIES

BTX therapy of chronic anal fissures has been performed for more than ten years now and a number of mostly single-centre studies have been published [Table 3]. The healing rates (≤ 6 months) are 60–90% and partial responses are seen in many patients. However, nonresponders are rare (< 5%) resulting mostly due to incorrect technique and/or diagnosis. The earliest effect is the antipruritic effect. It is important to note that fissure healing often takes more time than pain relief. Long-term outcomes (>12 months) show complete healing rates of about 50%.[8]

**Table 3:** Selection of internationally published studies using botox in chronic anal fissure

Author(s)	Year	No. of patients	Units/ Injection into*	Complete response (%)
Gui *et al*.[14]	1994	10	15 B/IAS	90
Jost and Schimrigk[15]	1994	12	5 B/EAS	83
Jost *et al*.[16]	1995	54	5 B/EAS	78
Jost[17]	1997	100	2.5 B/EAS	82
Maria *et al*.[18]	1998	15	20 B/ISA	73
		15	NaCl	13
Maria *et al*.[19]	1998	23	15 B/IAS	100
		34	20 B/IAS	100
Minguez *et al*.[20]	1999	23	10 B/IAS	83
		27	15 B/IAS	78
		19	21 B/IAS	90
Brisinda *et al*.[21]	1999	25	20 B/IAS	96
		25	Nitroglycerin	60
Fernandez *et al*.[22]	1999	76	40 B/IAS	67
Maria *et al*.[9]	2000	25	20 B/IAS posterior	80
		25	20 B/IAS anterior	100
Lysy *et al*.[23]	2001	15	20 B/IAS	73
			+ Nitroglycerin	
		15	20 B/IAS	60
Madalinski *et al*.[24]	2001	14	25–50 B/EAS	54
Brisinda *et al*.[25]	2002	75	20 B (+20)/IAS	89
		75	30 B (+50)/IAS	96
Trcinski *et al*.[26]	2002	13	100 D/IAS	85
Colak *et al*.[27]	2002	3	30 B/IAS	71
		28	Lidocain	21
Wollina *et al*.[4]	2002	5	20–25 B/EAS	40
		5	20–25 B/EAS	100
			+ 30–50 B for hyperhidrosis	
Mentes *et al*.[28]	2003	61	20–30 B/IAS	87
		50	Sphincterotomy	98
Siproudhis *et al*.[29]	2003	22	100 B/IAS	32
		22	NaCl	32
Lindsey *et al*.[30]	2003	40	20 B/IAS	43
Giral *et al*.[31]	2004	10	30 B/IAS	70
		11	Sphincterotomy	82
Godevenos *et al*.[32]	2004	45	25 B/IAS	18
		37	2 × 25 B/IAS	78
Arroyo *et al*.[2]	2005	100	25 B/IAS	47
Arroyo *et al*.[33]	2005	40	25 B/IAS	45
		40	Sphincterotomy	92
Iswariah *et al*.[34]	2005	17	40 B/IAS	41
		21	Sphincterotomy	91
Massoud *et al*.[35]	2005	25	20 B/IAS	64
		25	Sphincterotomy	100
Kinney *et al*.[36]	2006	22	100 B/intraanal	91
Floyd *et al*.[37]	2006	32	60 B/IAS	63
Tranqui *et al*.[38]	2006	50	30–100 B/IAS	94
			+ Nifedipin	
Witte *et al*.[39]	2007	100	40–100 D/IAS	77
Radwan *et al*.[13]	2007	38	10–20 B/IAS	89

* IAS - internal anal sphincter; EAS - external anal sphincter; B - Botox®; D - Dysport®

### Complications

Faecal incontinence is a severe adverse effect of surgery. With BTX, mild temporary incontinence of flatus has been reported in ≤ 6% of the patients.[10] Abscess formation is very rare, whereas this adverse effect is seen in about 13% of lateral sphincterotomy patients.[11] Jost *et al*. reported a single case of perianal thrombosis after BTX injection into the external sphincter muscle.[12] Radwan *et al*. reported two patients with temporary faecal soiling after BTX injection into the internal sphincter muscle.[13]

### Comparison of BTX to other established treatments and combinations

The gold standard for the management of recalcitrant chronic anal fissures has been lateral sphincterotomy. In a retrospective evaluation of 562 chronic anal fissure patients, complete healing was achieved within 31 weeks compared to 56 weeks with BTX. The relapse rate in the BTX group was significantly higher (35%), with 7% eventually treated by surgery.[37]

In a meta-analysis, Sajid *et al*. compared surgical *vs* chemical sphincterotomy using BTX for the treatment of chronic anal fissures. Surgical sphincterotomy had a significantly higher healing rate [*P* < 0.0110] and a significantly lower recurrence rate [*P* < 0.0221] than BTX. However, there was a higher complication rate [*P* < 0.0163] and a higher risk of transient faecal incontinence [*P* < 0.0001] in the sphincterotomy group than in the BTX group.

Scholz *et al*. treated 40 patients with combined fissurectomy followed by injection of 10 U of Botox® into the internal anal sphincter on both sides of the fissure. At six weeks, 38 patients (95%) were free of symptoms and no adverse effects were detected. The response rate of questionnaires was 93%; the median follow-up was one year (0.9–1.6 years). In the long-term, recurrence was found in four patients who were treated successfully with repeated fissurectomy, BTX injections, and salvage procedures. The overall success rate of combined fissurectomy and BTX injection was 79%, this combined approach scoring as an excellent and safe procedure with low morbidity and a high healing rate for chronic fissures.[40]

Brisinda *et al*. evaluated the efficacy of BTX injection in the treatment of recurrent anal fissures following lateral internal sphincterotomy. Eighty patients were treated with botulinum toxin (30 U Botox® or 90 U Dysport®) injected into two sites of the internal sphincter. One month after injection, there was complete healing in 54 patients (68%). Eight patients (10%) reported mild incontinence of flatus that disappeared spontaneously within two months. At two months, 59 patients (74%) had a healing scar. Anorectal manometry at one month demonstrated a significant reduction in both resting anal pressure and maximum voluntary squeeze pressure (*P* < 0.001). There were no relapses during an average follow-up period of 57.9 months.[41]

These observations are further supported by a recent study of Baraza *et al*. in females, who have an increased risk of incontinence after surgery. The authors combined BTX injection for anal fissures with fissurectomy. Forty-six female patients who had undergone excision of the fissure edges and injection of 25–100 U of BTX (Botox®, Dysport®) into the intersphincteric space, were followed up two months after the procedure and over a period of up to 39 months. No patient had any postoperative incontinence symptoms. There was a cure rate of 85% in 44 patients at a median follow-up period of 11 months. Fissurectomy and botulinum toxin injection for the treatment of chronic anal fissure in females seems to be effective in the medium-term, but there is a high rate of late recurrence. However, only a minority of patients proceed to more invasive surgical intervention, which may make it a useful option in patients who are not suitable for lateral sphincterotomy.[42,43]

In a prospective, controlled, two-armed trial comparing 0.2% nitroglycerine nitrate ointment with 20 U Botox®, a complete healing was achieved in 66.7 or 57.1% of the patients, respectively after three months. Relapses were seen in 33% of the patients in both groups. After three years, complete healing was seen in 40.0 and 33.3% of the patients in the nitroglycerine and Botox arms, respectively.[44] The combination of nitroglycerine ointment and BTX seems to potentiate the healing effect.[45] The combination of nifedipin and BTX (30–100 U Botox®) resulted in a remarkable low relapse rate of 2% (*n* = 47).[38]

In a meta-analysis, Sajid *et al*. compared the effectiveness of BTX and glyceryltrinitrate (GTN) for the pharmacological management of chronic anal fissures (CAF). BTX and GTN were equally effective in healing/improving chronic anal fissures. GTN was associated with a higher incidence of total side effects [*P* = 0.0002], especially headache [*P* = 0.0007]. Their conclusion was that BTX is as effective as GTN for the management of chronic anal fissures, but that it is associated with a lower complication rate. BTX has therefore been recommended as a first-line therapy for chemical sphincterotomy.[46]

## GUIDELINES FOR THE PRACTICE

The management of patients with chronic anal fissures involves both general and local measures. The treatment needs experience and skills and should be carried out only by those specialized in proctology. In Europe, proctology is performed by dermatologists, gastroenterologists, and specialized surgeons.

First-line therapy is always conservative and involves using appropriate nutrition, fluid intake, and exercises. Such treatment is easy, cheap, and safe but this remains the basis for all other treatments as well.[1]

Among conservative medical procedures, diltiazem, nifedipin, and nitroglycerine can be considered as first-line treatments. However, the effect is delayed with these treatments, and nitroglycerine has a significant risk of headaches.[46] If there is no response by eight weeks, BTX injections should be considered.

BTX is a safe and effective treatment for anal fissures. The effect is dose-dependent and technique-dependent. Doses of 20–40 U Botox® or 50–100 U Dysport® are useful as long as the patient does not suffer from anal fold hyperhidrosis, in which case, higher doses may be necessary. Both techniques: injections along both sides of a chronic fissure or along the anterior midline, are effective. There is some debate about the site of injection, as both internal and external sphincters have their advocates as mentioned earlier. The injection into the internal sphincter is supported by the pathophysiology of anal fissures, but is more difficult to give and more painful. Injection into the external sphincter is easier and less painful.[8] Combination of BTX with either nitroglycerine or nifedipin reduces relapse rates. The role of fissurectomy in conjuction with BTX needs further investigation.[47] Using this algorithm, lateral sphincterotomy can be avoided in most cases and BTX can also be used in patients with a relapse after surgical sphincterotomy.

In summary, BTX is a useful and safe alternative in the management of anal fissures. BTX does not cause any downtime for the patient and is more convenient than surgery. However, in some patients, particularly those with underlying pathologies, BTX may not be adequate and such patients may eventually need sphincterotomy.
